# Brain functional mechanisms of acupuncture treatment for early-stage non-tremor Parkinson’s disease: a resting-state fMRI study

**DOI:** 10.3389/fneur.2026.1924955

**Published:** 2026-09-29

**Authors:** Yanjin Qin, Runni Liu, Rujun Liu

**Affiliations:** 1 Faculty of Chinese Medicine, Macau University of Science and Technology, Macau, Macao SAR, China; 2 Department of Neurology, Zhuhai Hospital of Integrated Traditional Chinese and Western Medicine, Zhuhai, Guangdong, China; 3 Department of Medical Imaging, Zhuhai Hospital of Integrated Traditional Chinese and Western Medicine, Zhuhai, Guangdong, China

**Keywords:** acupuncture, non-tremor, observational study, Parkinson’s disease, resting-state functional magnetic resonance imaging

## Abstract

**Objective:**

To observe the modulatory effects of acupuncture in early-stage non-tremor Parkinson’s disease (PD) patients using resting-state functional magnetic resonance imaging (rs-fMRI), and to determine the correlation between its changes and clinical indicators.

**Methods:**

This observational study included 35 patients with early Parkinson’s disease who received multi-target acupuncture treatment combined with regular levodopa therapy (twice weekly for 12 weeks) in the traditional Chinese medicine department. They were classified as the observation group and the study period was from January 2023 to December 2024. The observation group received acupuncture and underwent rs-fMRI scanning at baseline and post-treatment. A healthy control group underwent a single rs-fMRI scan at baseline. Post-treatment imaging and clinical data from the observation group were compared with those of the healthy control group. Primary outcomes were changes in rs-fMRI-derived degree centrality (DC), functional connectivity (FC), and fractional amplitude of low-frequency fluctuations (fALFF). Secondary outcomes included clinical scale improvements and correlations between imaging metrics and clinical scores.

**Results:**

After treatment, the observation group showed significant improvements in MDS-UPDRS-III, MoCA, 10MWT, TUG, and PDQ-39 scores (all *p* < 0.001). However, all indicators except MoCA remained significantly different after treatment (*p* < 0.001). fMRI analyses revealed significant post-treatment decreases in right precuneus DC and its FC with the left precuneus (both *p* < 0.001), the fALFF in medial superior frontal gyrus/anterior cingulate cortex increased(*p* < 0.001). The change (post-treatment minus pre-treatment) in right precuneus DC was positively correlated with the change (post-treatment minus pre-treatment) in MDS-UPDRS-III scores (r = 0.343, *p* = 0.043) and negatively correlated with the change (post-treatment minus pre-treatment) in MoCA scores (r = −0.533, *p* < 0.001).

**Conclusion:**

Acupuncture treatment was associated with improvements in motor function, walking speed, and cognition in early-stage non-tremor PD, with potential mechanisms involving downregulated precuneus DC/FC and enhanced local activity. Furthermore, the change in right precuneus DC, which correlated significantly with both motor and cognitive improvements, may serve as a potential neuroimaging biomarker for treatment-induced clinical recovery.

## Introduction

Parkinson’s disease (PD) prevalence increasing annually due to population aging, placing a heavy burden on both society and families (1, 2). PD can be classified into tremor-dominant (TD) and non-tremor subtypes, the latter primarily characterized by PIGD (3). PIGD experience faster disease progression, higher risk of falls, more pronounced cognitive decline, and poorer response to conventional dopaminergic therapy (4, 5). Therefore, identifying effective adjunctive treatments specifically for the non-tremor subtype is of significant clinical importance.

Acupuncture has been consistently supported by multiple clinical studies for its efficacy in alleviating symptoms in patients with PD (6, 7). These symptomatic improvements include enhancements in gait and balance, as well as relief from autonomic dysfunctions such as constipation and sleep disturbances (6, 7). Despite these encouraging clinical findings, most existing functional magnetic resonance imaging (fMRI) investigations into the neural mechanisms of acupuncture have predominantly targeted patients with mixed-type or TD of PD. Consequently, research specifically examining brain functional network plasticity in the non-tremor subtype remains notably limited (8, 9).

Accumulating evidence from resting-state fMRI (rs-fMRI) studies has revealed widespread functional connectivity abnormalities across several large-scale brain networks in PD patients. These alterations involve not only the sensorimotor network but also higher-order networks, which are critical for motor coordination and cognitive processing (10, 11). Intriguingly, a recent study demonstrated that acupuncture at the acupoint Yanglingquan (GB34) significantly reduced spontaneous neural activity in the occipital lobe of PD patients—an effect that was absent in healthy controls. This finding suggests that the neuromodulatory effects of acupuncture are disease-state dependent, meaning they may specifically act upon aberrant neural activity patterns associated with the pathological state rather than inducing uniform effects across different populations (12). Such disease-specific modulation may offer critical clues for identifying neural targets for acupuncture interventions tailored to the PIGD-dominant non-tremor subtype, a group that remains understudied in the neuroimaging literature.

Despite the aforementioned cross-sectional evidence, longitudinal fMRI studies observing the functional network plasticity in patients with the non-tremor subtype are still lacking. In particular, dynamic change analyses based on baseline references from healthy controls are even more scarce. Therefore, the present study adopted a longitudinal observational design and included a healthy control group to provide baseline references, aiming to systematically elucidate resting-state brain functional networks in early-stage non-tremor PD, thereby providing neuroimaging evidence for acupuncture as an adjunctive treatment.

## Methods

### Study design

This observational study included 35 patients with early-stage non-tremor PD who received conventional anti-Parkinsonian medication combined with multi-target acupuncture treatment (twice weekly for 12 weeks). The observation group underwent rs-fMRI scanning at baseline and post-treatment. A healthy control group consisting of 35 age- and gender-matched healthy volunteers underwent baseline assessment only. The study period was from January 2023 to December 2024. The study protocol was approved by the Ethics Committee of Zhuhai Hospital of Integrated Traditional Chinese and Western Medicine (Approval Number: 20220513002).

### Participants

#### Inclusion criteria for the observation group

(1) Age: 40–80 years old, right-handed; (2) Patients with primary PD who meet the clinical diagnostic criteria of MDS 2015 (13); (3) Hoehn-Yahr stage 1–2.5; (4) Disease duration ≤5 years; (5) Non-tremor subtype classification (14): According to the third part of MDS-UPDRS, using the TD/PIGD ratio method, the PIGD score≥1.15 and the TD score<PIGD score; MoCA≥24 points; (6) The dosage of anti-PD drugs has been stable for the past 4 weeks.

#### Exclusion criteria for the observation group

(1) Atypical Parkinson’s syndrome or secondary Parkinson’s syndrome; (2) H&Y stage ≥ 3; (3) Having MRI contraindications (such as cardiac pacemaker, intracranial metal implants, claustrophobia, etc.); (4) Having received acupuncture treatment before or being afraid of/ experiencing acupuncture-induced syncope; (5) Having skin infection or ulcer at the acupuncture site; (6) Having severe dysfunction of heart, liver, or kidney or mental illness; (7) Pregnant or breastfeeding; (8) Participating in other clinical trials simultaneously.

#### Inclusion criteria for the healthy control group

(1) Age and gender are matched with those of the observation group (age difference between groups ≤ 5 years), right-handed; (2) No history of any neurological diseases or mental disorders; (3) No contraindications for MRI; (4) MMSE score≥27; (5) Voluntary to sign the informed consent form.

### Sample size estimation

Based on the effect size from previous fMRI studies on acupuncture (15) (Cohen’s *d* ≈ 0.8–1.0), with α = 0.05 (two-sided), and a power of 1-β = 0.80, the sample size was estimated using the paired *t*-test method. The required sample size for the paired *t*-test is 20 cases; considering a 20% dropout rate, each group needs at least 24 cases; to ensure the feasibility of the statistical power, and referring to the sample size settings of similar observational studies, the observation group in this study included 35 cases, and the healthy control group included 35 cases. The total sample size: 70 cases.

### Treatment plan

#### Acupuncture therapy

Based on traditional Chinese medicine theory and clinical literature reports (16), in this study, the following acupoints were selected: Baihui (GV20, needled horizontally 15–20 mm, after feeling the qi, rotate the needle), Yintang (EX-HN3, pinch and insert the needle horizontally 10–15 mm), Fengfu (GV16, needled directly 15–20 mm, pay attention to the direction and depth), bilateral Hegu (LI4, needled directly 20–25 mm, lift and rotate the needle), bilateral Taichong (LR3, needled directly 15–20 mm, lift and rotate the needle), bilateral Yanglingquan (GB34, needled directly 20–30 mm, lift and rotate the needle), and bilateral Zusanli (ST36, needled directly 25–30 mm, lift and rotate the needle). See Figure 1. All acupoints were treated with disposable sterile acupuncture needles. The deqi sensation (a composite of soreness, numbness, heaviness, and distension) was assessed subjectively by each patient’s self-report immediately after needle manipulation, following standard acupuncture methodology (17, 18). The needles were left in for 30 min, and during this period, a simple reinforcing and counter-reducing technique was performed once every 10 min. The treatment frequency is twice a week, and the treatment course lasts for 12 weeks. The interval between each treatment and medication administration was more than 1 h. The treatment was performed by the same acupuncturist with ≥5 years of clinical experience.

**Figure 1:**
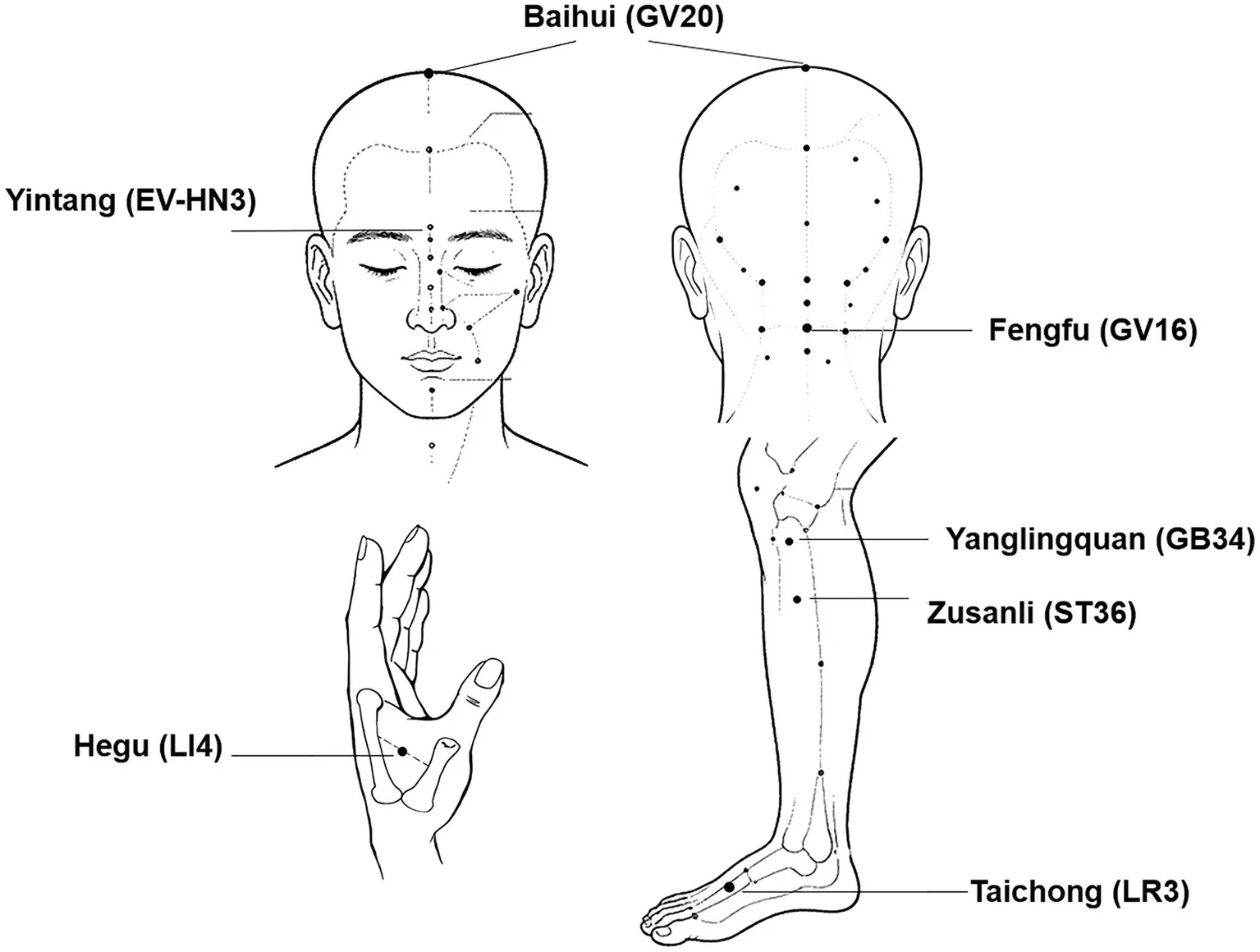
Acupuncture meridian map.

#### Basic drug therapy

For patients with PD in the observation group, the original anti-PD drugs were continued, and the dosage was maintained stable throughout the entire observation period.

#### Record of adverse events

The following adverse events were recorded: needle syncope, needle stasis, needle breakage, subcutaneous hematoma, local infection, and increased pain. Severe adverse events were reported to the ethics committee in accordance with relevant regulations.

### Resting-state fMRI data acquisition

Participants were instructed to lie supine with their eyes closed, remain awake, and avoid systematic thinking during scanning. Noise-canceling earplugs and a foam pillow were provided to minimize head motion and scanner noise. All participants underwent a 5-min acclimatization period in the scanning environment before data acquisition. Whole-brain images were acquired in the axial and sagittal planes.

MRI data were acquired using a Siemens 3.0 T MAGNETOM Skyra scanner (Siemens Healthcare, Erlangen, Germany). High-resolution T1-weighted anatomical images were obtained using a magnetization-prepared rapid acquisition gradient-echo (MPRAGE) sequence with the following parameters: TR = 1900 ms, TE = 2.53 ms, TI = 900 ms, flip angle = 9°, FOV = 250 mm × 250 mm, matrix = 256 × 256, slice thickness = 1.0 mm, 192 sagittal slices, scan duration = 366 s. EPI sequence params: FOV = 192 mm × 192 mm, TR = 3,000 ms, TE = 30 ms, number of slices = 36, slice thickness = 3.0 mm, slice gap = 0.75 mm, flip angle = 90°, matrix = 64 × 64, NEX = 1, scan duration = 608 s.

All imaging data were preprocessed and analyzed using SPM12 (Statistical Parametric Mapping, Wellcome Centre for Human Neuroimaging, London, UK) and DPABI (Data Processing & Analysis for Brain Imaging), running on MATLAB R2018b (MathWorks, Natick, MA, USA). Spatial normalization to the MNI152 standard space was performed via the DARTEL (Diffeomorphic Anatomical Registration Through Exponentiated Lie Algebra) algorithm implemented in SPM12, using each subject’s T1-weighted image to generate normalization parameters, which were then applied to the co-registered functional images. After normalization, functional images were resampled into 3 mm isotropic voxels and spatially smoothed with a 4 mm FWHM Gaussian kernel.

Based on these preprocessing steps, the following metrics were extracted: (1) Degree centrality (DC): whole-brain voxel-wise DC analysis (threshold *r* > 0.2) was performed, and DC values were extracted from regions of interest including the striato-thalamo-cortical (STC) circuit (bilateral putamen, thalamus), default mode network (DMN; bilateral precuneus, medial prefrontal cortex), and sensorimotor network (SMN; supplementary motor area, primary motor and sensory cortices). These ROIs were selected based on their established roles in PD pathophysiology and acupuncture modulation (19). (2) Functional connectivity (FC): Seed-based FC analyses were conducted using both ROI-based and voxel-based approaches. Using the bilateral precuneus [MNI coordinates: ±3, −54, 63; based on its role as the DMN hub (20)], putamen [±24, 6, 6; core component of the basal ganglia-thalamo-cortical motor circuit (21)], thalamus [±12, −15, 9; key relay nucleus in motor and cognitive circuits (22)], supplementary motor area [±6, −6, 54; critical for motor planning and initiation (23)], and cerebellar dentate nucleus [±24, −54, −36; involved in cerebro-cerebellar motor coordination (21)] as seed regions, FC values between each seed and whole-brain voxels were calculated. (3) Fractional amplitude of low-frequency fluctuations (fALFF): fALFF values were extracted from both predefined ROIs and via whole-brain voxel-wise analysis. For all three metrics, both ROI-based and voxel-based whole-brain analyses were performed. All metric extraction and data processing procedures were independently performed and confirmed by two researchers with over 3 years of experience in fMRI data analysis to ensure the reliability of the results.

### Clinical assessment tools

This study employed the following clinical assessment tools. All scales were evaluated before and after treatment: The total score of MDS-UPDRS-III ranged from 0 to 132, with a lower score indicating improved motor symptoms (24); The Berg Balance Scale (BBS) consisted of 14 items, with a score ranging from 0 to 56, and an increase in the score reflected enhanced balance function and reduced risk of falling (25); The 10-meter Walking Test (10MWT) recorded the walking speed in meters per second, and an increase in speed indicated improved walking ability (25); The Timed Up and Go Test (TUG) recorded the time taken to complete the “stand-up-walk-return” (in seconds), and a shorter time indicated improved mobility and reduced risk of falling (25); The Montreal Cognitive Assessment (MoCA) ranged from 0 to 30, and an increase in the score suggested improved cognitive function (26); The Parkinson’s Disease Quality of Life Questionnaire (PDQ-39) had a total score ranging from 0 to 100, and a lower score indicated improved quality of life (27). The healthy control group underwent a single baseline assessment using the same clinical scales and MRI protocol. This served to provide normative reference values for cross-sectional comparisons with the observation group at enrollment, allowing us to characterize the baseline functional and clinical impairments in early-stage non-tremor PD patients (Figure 2).

**Figure 2:**
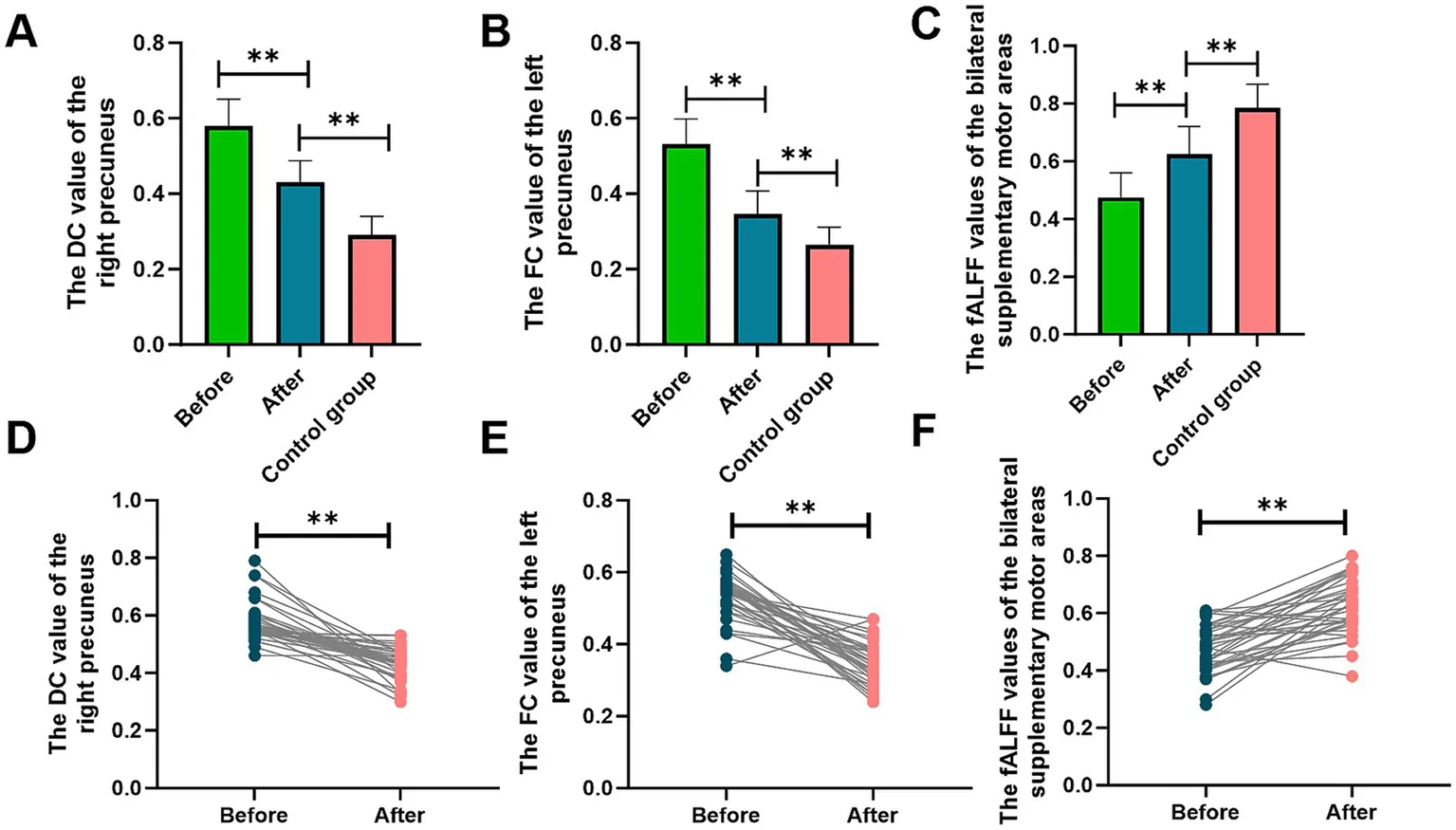
Comparison of fMRI indicators before and after treatment in the observation group. **(A–C)** Show group mean values with error bars (mean ± SD) before and after treatment; **(D–F)** show individual subject trajectories (line plots) with paired *t*-test results for each imaging metric. **p < 0.001.

### Statistical analysis

Clinical data were analyzed using SPSS 26.0 software. Continuous variables following a normal distribution were expressed as mean ± standard deviation (SD), and Mann–Whitney *U* tests or Wilcoxon signed-rank tests were applied as appropriate. Correlation analyses were performed using Pearson correlation for normally distributed data and Spearman correlation for non-normally distributed data. Comparisons between the observation group at baseline and the healthy control group were conducted using two-sample *t*-tests. Changes in clinical and imaging variables were defined as post-treatment value minus pre-treatment value for all analyses. For fMRI data, ROI-based analyses used paired *t*-tests; voxel-based whole-brain analyses used voxel-wise paired *t*-tests comparing post-treatment versus pre-treatment, performed using DPABI^1^ and SPM12 (Wellcome Department of Imaging Neuroscience, London, UK) running on MATLAB R2018b. Head motion was assessed by calculating framewise displacement (FD) using the Jenkinson formula. No subjects were excluded based on the pre-established motion criteria (>2.0 mm translation or >2.0° rotation). Motion scrubbing was not applied; instead, the Friston-24 head motion parameters were included as covariates in the general linear model to regress out motion-related variance. Age, sex, and mean framewise displacement (FD) were included as covariates in the general linear model for between-group comparisons. All fMRI results were corrected for multiple comparisons using Gaussian Random Field (GRF) theory, with a voxel-level threshold of *p* < 0.005 and a cluster-level threshold of *p* < 0.05 (two-tailed).

## Results

### Comparison of patient general information

There were no differences in gender, age, BMI, education years, past medical history, family history, smoking history, or allergy history (*p* > 0.05). See Table 1.

**Table 1:** Comparison of General Information.

General information	Control group (*n* = 35)	Observation group (*n* = 35)	*t/* $\chi^{2}$	*P*
Gender/*n* (%)			0.230	0.631
Male	20 (57.14)	18 (51.43)		
Female	15 (42.86)	17 (48.57)		
Age, (years, $\bar{x\;}\pm s$ )	66.11 ± 11.50	65.03 ± 9.61	0.422	0.674
BMI (kg/m^2^, $\bar{x}\pm s$ )	20.88 ± 2.06	21.21 ± 2.06	−0.674	0.503
Education years, (years, $\bar{x}\pm s$ )	9.46 ± 1.66	9.31 ± 2.09	0.312	0.756
Past medical history/*n* (%)			0.583	0.445
Yes	10 (28.57)	13 (37.14)		
No	25 (71.43)	22 (62.86)		
Family history/*n* (%)			0.108	0.743
Yes	6 (17.14)	5 (14.29)		
No	29 (82.86)	30 (85.71)		
Smoking history/*n* (%)			0.058	0.810
Yes	15 (42.86)	16 (45.71)		
No	20 (57.14)	19 (54.29)		
Allergy history/*n* (%)			0.233	0.629
Yes	21 (60.00)	19 (54.29)		
No	14 (40.00)	16 (45.71)		

Data are presented as mean ± standard deviation ( $\bar{x}\pm s$ ) for normally distributed continuous variables, *n* (%). *t*-values are from independent two-sample *t*-tests; χ^2^ values are from chi-square tests. *p* < 0.05 was considered statistically significant.

### Comparison of motor function scores

After treatment, MDS-UPDRS-III score of the observation group decreased from 62 (49, 71) to 49 (40, 56), representing a median reduction of 13 points (*p* < 0.001), and the MoCA score significantly increased from 25 (24, 26) to 28 (27, 29), representing a median improvement of 3 points (*p* < 0.001), while the BBS score showed an upward trend but did not reach statistical significance (*p* = 0.153). The comparison between the groups revealed that, except for the MoCA score which was close to that of the healthy group, there were still differences in all indicators between the observation group after treatment and the healthy group (*^a^p* < 0.001). See Table 2.

**Table 2:** Comparison of motor function scores [M (Q_25_, Q_75_)].

Indicator	Control group (*n* = 35)	Observation group (*n* = 35)		*Z*	*P*
		Before	After		
MDS-UPDRS-III (score)	0 (0, 0)	62 (49, 71)	49 (40, 56)^a^	−3.820	<0.001
BBS (score)	54 (54, 55)	50 (48, 52)	51 (50, 52)^a^	−1.429	0.153
MoCA (score)	28 (28, 29)	25 (24, 26)	28 (27, 29)	−6.628	<0.001

Data are presented as median (Q_25_, Q_75_). *Z* values are from Wilcoxon signed-rank tests for within-group comparisons between baseline and post-treatment in the observation group; Compared with the Control group, ^a^*P* < 0.001.

### Comparison of walking speed

The 10MWT significantly increased from (0.90 ± 0.05) m/s to (1.15 ± 0.05) m/s (an increase of 0.25 m/s, +27.8%) after treatment (*p* < 0.001), the TUG significantly shortened from (16.10 ± 1.26) s to (12.84 ± 1.34) s (a reduction of 3.26 s, −20.2%) (*p* < 0.001), and the PDQ-39 score significantly decreased from 32 (27, 28) to 24 (20, 26) (a median reduction of 8 points, −25.0%) (*p* < 0.001). The comparison between the groups showed that there were still significant differences in all indicators between the observation group and the health group after treatment (*^a^p* < 0.001). See Table 3.

**Table 3:** Comparison of walking speed and quality of life.

Indicator	Control group (*n* = 35)	Observation group (*n* = 35)		*t/Z*	*P*
		Before	After		
10MWT (m/s, $\bar{x}\pm s$ )	1.29 ± 0.09	0.90 ± 0.05	1.15 ± 0.05^a^	−21.599	<0.001
TUG (s, $\bar{x}\pm s$ )	9.03 ± 1.01	16.10 ± 1.26	12.84 ± 1.34^a^	10.335	<0.001
PDQ-39 [M (Q_25_, Q_75_)]	0 (0, 0)	32 (27, 39)	24 (20, 26)^a^	−4.552	<0.001

Data are presented as mean ± standard deviation ( $\bar{x\;}\pm s$ ) (10MWT, TUG) or median (Q25, Q75) (PDQ-39). *t* values are from paired *t*-tests and *Z* values are from Wilcoxon signed-rank tests for within-group comparisons between baseline and post-treatment in the observation group; Compared with the Control group, ^a^*P* < 0.001.

### Incidence of adverse reactions during treatment

During the treatment period, there were 2 cases of subcutaneous hematoma and 1 case of local infection in the observation group. After treatment, the symptoms all subsided and did not affect the treatment process.

### Comparison of brain regions before and after treatment in the observation group

ROI-based analyses showed that after treatment, the DC value of the right precuneus significantly decreased (*p* < 0.001); using the right precuneus as the seed point, the FC between it and the left precuneus also significantly weakened (*P* < 0.001); while the fALFF values in the bilateral supplementary motor area/medial superior frontal gyrus/anterior cingulate cortex (BA 8/32) significantly increased (*P* < 0.001) (Table 4).

**Table 4:** Comparison of brain regions before and after treatment.

Indicator	Dissimilar brain regions	BA	MNI coordinates (mm)			Cluster size (voxels)	*t*
			*x*	*y*	*z*		
DC	Right precuneus	BA5_R	12	−51	57	45/27	−10.11^b^
FC	Left precuneus (seed point: right precuneus)	BA7_R	−3	−66	51	124/50	−14.97^b^
fALFF	Bilateral supplementary motor area/medial frontal superior gyrus/left anterior cingulate/lateral cingulate gyrus	BA 8/32	−12	48	21	168/79	9.04^b^

BA = Brodmann area; R in BA7_R indicates that the cluster is located predominantly in the right hemisphere. The fALFF region (BA 8/32) comprises bilateral clusters (including bilateral supplementary motor area, medial superior frontal gyrus, and anterior cingulate cortex). GRF correction, voxel level *p* < 0.005, cluster level *p* < 0.05. Cluster size is presented as “total voxels in the cluster/voxels in the primary sub-cluster containing the peak voxel”. Each voxel is 3 × 3 × 3 mm (isotropic, volume = 27 mm^3^). The paired *t*-test correction was based on *n* = 35, *r* = 0.5, ^b^*P* < 0.001.

Voxel-based whole-brain analyses were further performed to complement the ROI-based findings. Consistent with the ROI results, paired t-tests revealed significant post-treatment decreases in right precuneus DC and decreased FC between the right and left precuneus, as well as increased fALFF in the bilateral supplementary motor area/medial superior frontal gyrus/anterior cingulate cortex (BA 8/32). No other brain regions showed significant differences in the whole-brain analysis (all GRF-corrected, voxel *p* < 0.005, cluster *p* < 0.05). The specific locations of these brain regions are presented in Figure 3 and detailed in Table 4.

**Figure 3:**
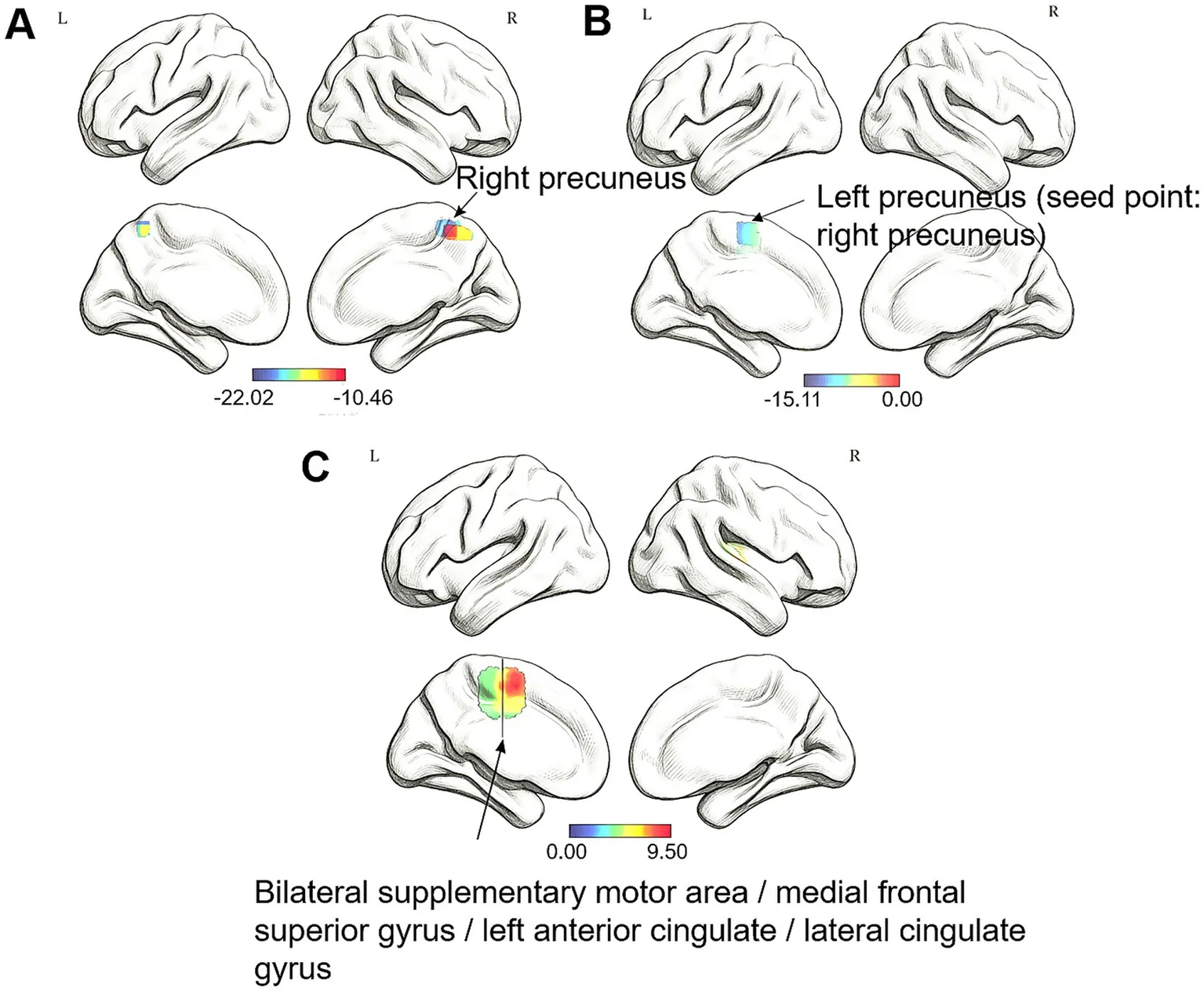
Illustration of brain regions showing differences before and after treatment based on voxel-based whole-brain analysis. **(A)** Differential brain regions of DC (right precuneus). **(B)** Differential brain regions of FC using the right precuneus as the seed (left precuneus). **(C)** Differential brain regions of fALFF (bilateral supplementary motor area/medial superior frontal gyrus/anterior cingulate cortex, BA 8/32). All results are derived from voxel-based whole-brain analyses (GRF-corrected, voxel *p* < 0.005, cluster *p* < 0.05) and displayed on a standard MNI brain template. The color bar represents *t*-values ranging from low (blue) to high (red).

### Correlation analysis of the right precuneus DC difference with changes in clinical scores

The correlation analysis of the differences in imaging indicators and clinical scores before and after treatment in the observation group showed that the change (post-treatment minus pre-treatment) in right precuneus DC was significantly positively correlated with the change (post-treatment minus pre-treatment) in MDS-UPDRS-III score (*r* = 0.343, *p* = 0.043), and was significantly negatively correlated with the change (post-treatment minus pre-treatment) in MoCA score (*r* = −0.533, *p* < 0.001). The differences in other FC and fALFF brain regions before and after treatment had no significant correlation with other clinical scores (such as BBS, PDQ-39, etc.) (*p* > 0.05). See Figure 4.

**Figure 4:**
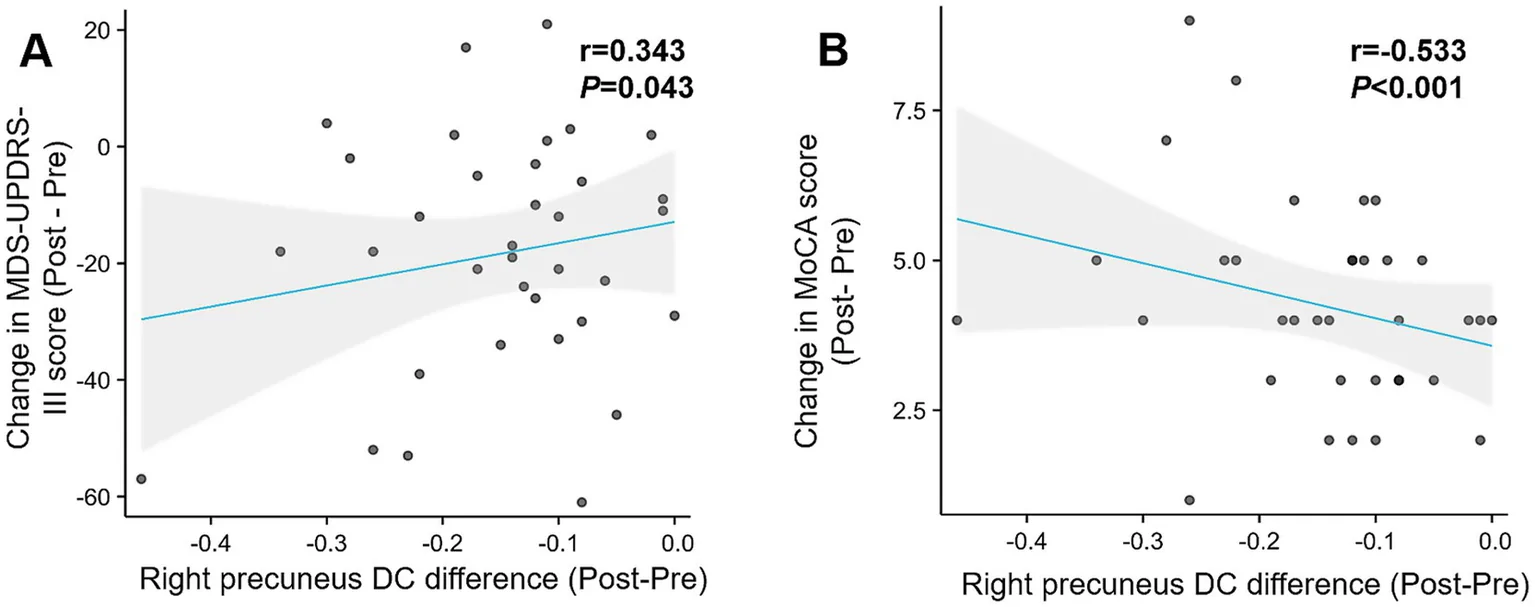
Correlations between treatment-related changes in the right precuneus DC and changes in clinical score. Changes are defined as the differences between post-treatment values and pre-treatment values (i.e., Post-Pre). **(A)** Correlation between changes in right precuneus DC and changes in MDS-UPDRS-III scores (*r* = 0.343, *P* = 0.043). **(B)** Correlation between changes in right precuneus DC and changes in MoCA scores (*r* = −0.533, *P* < 0.001).

## Discussion

The main clinical feature of PD is motor dysfunction (29). The disease severely impacts patients’ quality of life, and conventional pharmacotherapy is often limited by insufficient efficacy and adverse effects (30). Acupuncture, as a non-pharmacological therapy, has unique advantages in improving both motor and non-motor symptoms of PD (31). In this study, we employed resting-state fMRI combined with multi-modal metrics including DC, FC, and fALFF to comprehensively analyze brain functional changes and clinical efficacy in patients with early-stage non-tremor PD before and after acupuncture treatment. We observed significant clinical improvements accompanied by decreased right precuneus DC/FC and increased fALFF in motor initiation regions. Correlation analysis further revealed that changes in right precuneus DC were significantly correlated with clinical improvements, suggesting that acupuncture may modulate brain network topology and local spontaneous activity to improve motor and cognitive function in early-stage PD.

MDS-UPDRS-III is an internationally standardized tool for assessing motor symptoms in patients with PD, and is widely used to monitor the efficacy of both pharmacological and non-pharmacological interventions. Its motor subscale specifically reflects core motor impairments (32). In this study, patients served as their own controls during the period of medication alone versus medication combined with acupuncture treatment. The results showed a significant reduction in MDS-UPDRS-III scores after combined treatment, indicating that adding acupuncture to conventional Western medication further improves overall motor function in patients with early-stage non-tremor PD. This finding is highly consistent with previous evidence-based medicine. One analysis found that acupuncture combined with conventional medication showed additional benefits across MDS-UPDRS-III domains compared with medication alone (31).

In PD, the MoCA is widely used to screen and assess PD-related cognitive deficits (33). In this study, the observation group showed an increase in MoCA scores after combined acupuncture treatment. A meta-analysis published in 2025, which included 9 randomized controlled trials involving 629 PD patients with cognitive impairment, demonstrated that acupuncture was significantly superior to conventional treatment controls in terms of MoCA scores, MMSE scores, and overall response rate (34). This may be attributed to acupuncture’s ability to bidirectionally regulate functional activity within the basal ganglia-thalamo-cortical circuit and promote neuroplastic changes in dopaminergic pathways, thereby improving bradykinesia and motor initiation difficulties. Acupuncture-induced functional changes in motor-related brain regions, including the precentral gyrus, putamen, thalamus, and cerebellum, support its modulatory effects via basal ganglia and cerebellar-cortical pathways (21). In summary, the improvements in MDS-UPDRS-III and MoCA scores observed in this study are mutually corroborated by clinical evidence in recent years, supporting acupuncture as a complementary therapy to conventional Western medication for comprehensively alleviating both motor and cognitive clinical symptoms in patients with early-stage non-tremor PD.

The 10MWT and TUG are objective quantitative measures for assessing walking ability and dynamic balance function in PD patients, and are closely associated with fall risk and activities of daily living (35, 36). The PDQ-39 is a PD-specific quality of life scale covering multiple domains including motor function, daily activities, and emotional wellbeing (37). In this study, after treatment, the observation group showed an increase in 10MWT, a reduction in TUG time, and a decrease in PDQ-39 scores from 32 (27, 39) to 24 (20, 26). This result is highly consistent with previously published clinical studies. A trial found that electroacupuncture significantly increased gait speed by 9 to 19% and stride length (38). Together, these findings support acupuncture as an adjunctive therapy with high clinical value for improving walking function and quality of life in patients with early-stage PD.

DC is a graph theory-based whole-brain network analysis metric used to identify core hub nodes within the brain’s functional network and to measure the information integration capacity and importance of a given brain region within the whole-brain network. In this study, the observation group showed a significant decrease in DC values in the right precuneus after treatment, indicating that the hub status of this brain region within the whole-brain network declined and its level of information integration weakened following acupuncture treatment. Abnormal precuneus function in PD has been extensively reported in the literature. One study found abnormally elevated DC values in bilateral precuneus and other brain regions in patients with PD-MCI (39). Another study also demonstrated that acupuncture-induced neural modulation is disease-state dependent, with significant effects observed in PD patients but not in healthy controls (17). This may be attributed to the precuneus is in a state of compensatory hyperactivation, serving to maintain normal motor initiation and cognitive processing functions. Acupuncture may help the brain network transition from an inefficient compensatory state to a physiological functional mode by downregulating the excessive integrative role of this node.

Functional connectivity analysis is used to measure the synchrony of time series activities between different brain regions, reflecting the degree of functional synergy between these brain regions. Functional connectivity analysis based on seed points can systematically evaluate the communication efficiency between core nodes and other regions of the whole brain without any prior assumptions (20). In this study, the FC value between Right precuneus and Left precuneus significantly weakened after treatment, suggesting that the synchrony between bilateral precuneus decreased after acupuncture treatment, and the abnormal information communication within the DMN declined. The DMN is highly active in the resting state and participates in advanced cognitive processes such as episodic memory, self-reference, and internal mental activities (28). The regulatory effect of acupuncture on precuneus has been verified in various neurological diseases. Precuneus activity changes under acupuncture stimulation have been correlated with balance ability in PD patients (12), and animal studies have demonstrated that acupuncture normalizes functional connections in PD models (40). We speculate that early PD patients may have abnormally enhanced synchrony between bilateral precuneus, which may excessively invade the motor network and interfere with motor initiation. Acupuncture may inhibit this excessive synchronous activity within the DMN, optimizing network resource allocation and improving motor initiation.

ALFF reflects the intensity of spontaneous neural activity in local brain regions within the low-frequency oscillation range and is a commonly used metric for measuring spontaneous neuronal activity, revealing local changes in the functional status of specific brain regions following acupuncture intervention (18). In this study, the observation group showed an increase in fALFF values in the bilateral supplementary motor area/medial superior frontal gyrus/left anterior cingulate gyrus/left paracingulate gyrus (BA 8/32) after treatment, indicating that acupuncture significantly enhances local spontaneous neuronal activity. The supplementary motor area (SMA) is critical for motor initiation and planning, and its reduced activity in PD is considered a key neural mechanism of akinesia (41). One study using scalp acupuncture to treat PD patients found that fALFF significantly increased in the left middle frontal gyrus after treatment, while DC increased in the left cerebellum, and there was a positive correlation between right limb swing and the fALFF region (42). This may be attributed to increase local neuronal excitability in the supplementary motor area through indirect regulation of the dopaminergic pathway, thereby restoring the functional output of the striatum-thalamus-supplementary motor area pathway and enhancing the ability to initiate internally triggered voluntary movements. This may represent a core mechanism by which acupuncture improves motor function in patients with early-stage PD.

Correlation analysis revealed that the change in the DC value of the right precuneus (post-treatment minus pre-treatment) was significantly positively correlated with the change in MDS-UPDRS-III scores, and significantly negatively correlated with the change in MoCA scores. One study found that the DC value of the Left precuneus was significantly negatively correlated with the MoCA score (43). This correlation pattern suggests that normalization of precuneus activity may serve as a key neuroimaging biomarker for treatment response in PD. The FC and fALFF indicators failed to establish significant correlations with clinical scores, possibly reflecting that the imaging changes at the population level do not have a completely linear mapping relationship with the symptoms at the individual level. Larger sample size studies and stratified analyses are needed to further clarify these relationships.

However, this study has the following limitations. Firstly, the relatively small sample size (35 cases per group) and single-center design may limit statistical power and generalizability. Secondly, only short-term effects were observed without long-term follow-up, and the optimal intervention parameters have not been systematically verified. Thirdly, the absence of a sham control group limits our ability to attribute the observed changes specifically to acupuncture, as non-specific effects cannot be ruled out. Although all patients maintained a stable drug dosage during the observation period to control pharmacological confounding, the observed improvements may still partially reflect non-specific effects. Future studies will conduct large-sample randomized trials, set up a sham acupuncture control group and parallel groups with different intervention parameters, and extend the observation period to 6–12 months after treatment to further verify the specific efficacy, long-term effect, and optimal treatment plan of acupuncture.

In summary, this resting-state fMRI study evaluated the clinical efficacy and neural mechanisms of acupuncture as an adjunct to conventional medication in early non-tremor PD. Acupuncture significantly improved motor, cognitive, and walking functions, as well as quality of life. Neuroimaging changes included decreased right precuneus DC and bilateral precuneus FC, alongside increased fALFF in motor initiation regions (SMA/ACC). Changes in right precuneus DC showed exploratory correlations with clinical improvements, suggesting that acupuncture may modulate brain network topology and local spontaneous activity. Given the observational design and absence of a sham control, these findings should be considered preliminary and hypothesis-generating.

## Data Availability

The raw data supporting the conclusions of this article will be made available by the authors, without undue reservation.

## Footnotes

1
http://rfmri.org/DPABI
